# Optimization of fucoidan recovery by ultrasound-assisted enzymatic extraction from South African kelp, *Ecklonia maxima*

**DOI:** 10.1016/j.ultsonch.2023.106710

**Published:** 2023-11-29

**Authors:** Zwonaka Mapholi, Neill Jurgens Goosen

**Affiliations:** Department of Chemical Engineering, Stellenbosch University, Private Bag X1, Matieland, 7602 Stellenbosch, South Africa

**Keywords:** *Ecklonia maxima*, Fucoidan, Ultrasound-assisted extraction (UAE), Enzyme-assisted extraction (EAE), Ultrasound-assisted enzymatic extraction (UAEE)

## Abstract

Fucoidan, a sulphated polysaccharide, is found exclusively in brown seaweeds and has been reported to possess a wide range of biological functionalities. Fucoidans are found within the cell wall of brown seaweeds, which is composed of recalcitrant cellulose and hemicellulose. This hampers the recovery of fucoidans. In addition, fucoidans are found within a network of viscous hydrocolloids, alginates, further complicating their recovery. Traditionally, the hot water extraction method is used to recover fucoidans from brown seaweed, however, this is characterized by low yields and long extraction time. To combat these issues, several novel extraction technologies have been introduced, these include ultrasound-assisted extraction and enzyme-assisted extraction. Thus, the main aim of this study was to investigate and optimize fucoidan recovery from *Ecklonia maxima* based on ultrasound-assisted enzymatic extraction. The impact of temperature (40–65 °C), ultrasound intensity (0–118 W·cm^−2^), enzyme dosage (0–0.05 ml·g^−1^) and pH (4.5–6) on total dissolved, total carbohydrates and inorganic sulphates yields was studied. The application of ultrasound-assisted enzymatic extraction mainly improved the extraction of total carbohydrates. Ultrasound significantly improves the kinetics and extraction of fucoidan, but there was no merit when it was applied with enzymes. Results reveal that at optimized conditions, the fucoidan extracted 79.13 mg⋅g^−1^ (7.9 % w/w) of algal dry weight. The present study provides insight into the extraction potentials of enzyme-assisted extraction, ultrasound-assisted extraction, and ultrasound-assisted enzymatic extraction.

## Highlights

• Extraction of fucoidan by ultrasound-assisted extraction from *Ecklonia maxima.*
• Optimization of ultrasound-assisted enzymatic extraction through response surface methods and desirability.
• Improved extraction kinetics were observed for ultrasound-assisted enzymatic extraction for total carbohydrates.
• Ultrasound is an effective method for fucoidan extraction.
• Identified optimum conditions for fucoidan extraction: 58.75 °C, 88.75 W⋅cm^−2^, pH 6, with no eznymes added. .
• 79 mg fucoidan⋅g^−1^ dry seaweed was extracted.

## 1 Introduction

Seaweeds hold great potential as a sustainable feedstock for value-adding bioprocesses that extract unique, high-value compounds. These compounds include ω-3 polyunsaturated fatty acids, proteins and peptides, phenolic compounds, carotenoids, and polysaccharides. Brown seaweeds are particularly rich in high-value polysaccharides such as fucoidan, laminarin and alginates. These polysaccharides reportedly possess numerous biological functionalities like anti-cancer, anti-diabetic, antioxidant, anti-viral, anti-bacterial and anti-inflammatory activities [1], [2], [3], [4], [5], [6]. In South Africa, the endemic brown seaweed *Ecklonia maxima* is commercially exploited within the aquaculture and biostimulant industries, with a minor fraction of the harvest exported in a dried form for alginate extraction abroad [7]*.* In South Africa, the bioresource is well-regulated for the purposes of industrial utilisation, which, coupled with a high natural growth rate, makes this a potential raw material for recovery of industrially valuable polysaccharides.

There is a need to develop environmentally friendly and efficient extraction processes for the extraction of seaweed polysaccharides, which also preserve the native quality of the extracted compounds [8]. Conventionally, seaweed polysaccharides are extracted through aqueous extractions at different pH values, typically employing strong acids and bases or hydrothermal treatment for primary extraction, which may be coupled with organic solvents during downstream processing [9], [10], [11]. Although the aqueous extraction methods can be selective towards specific polysaccharides, drawbacks include high resource requirements by using large volumes of water and chemicals and employing long extraction times (sometimes at elevated temperatures), to obtain economical yields of target compounds. The use of large volumes of water during extraction complicates downstream processing such as drying [10], and processing at elevated temperatures for extended periods can compromise the quality and thus the functionalities of these polysaccharides [12]. Promising alternative extraction methods include ultrasound-assisted extraction (UAE), enzyme-assisted extraction (EAE), microwave-assisted extraction (MEA), supercritical fluid extraction (SFE) and subcritical water extraction (SWE) [13], [14], [15], [16], [17], [18], [19].

The use of EAE for polysaccharides from seaweed is an attractive option, which is attributed to the unique characteristics and advantages of enzymes. Enzymes can catalyze reactions with high specificity, they function under a mild aqueous environment which could preserve the quality of the extracts, and EAE is easily scalable, with favorable potential for industrial application. A large proportion of seaweed polysaccharides occur in the cell wall, where they are enclosed in a cellulose and hemicellulose cell wall matrix, where they are further associated with proteins and other storage polysaccharides. Enzymes can disrupt the cell wall and the associated proteins, which results in easier solvent access and thus dissolution of compounds from the cell wall. Various commercial enzymes have been used to extract polysaccharides from seaweed [8], [13], [15], including carbohydrases and proteases. Despite the merits of EAE, its application could be impeded by the high cost of enzymes, limited enzyme recycling opportunities and long extraction times [8]. Furthermore, there are no enzymes tailored specifically for seaweed hydrolysis, meaning that enzymes optimized for terrestrial biomass are used, and possibly at sub-optimal performances [10].

UAE is another attractive option to utilize for seaweed polysaccharides, with multiple potential advantages, including short extraction time, being energy-efficient, operating at milder processing conditions and yielding extracts with higher quality compared to conventional aqueous extracts. The application of ultrasound in a liquid medium during UAE results in acoustic cavitation and extraction is mainly attributed to the physical effects of acoustic cavitation: microstreaming, microjets and micro turbulences. Cavitation near solid plant matrices leads to surface peeling, erosion and microfractures which leads to porous cell matrices, facilitating solvent penetration. Cavitation can improve extraction efficiencies through overall improvement of mass transfer by accelerating eddy and internal diffusion [8], [20]. Ultrasound can be used as a stand-alone extraction technique [21], [22], [23], and it can also be integrated and combined with other extraction techniques to further improve extraction efficiencies. Combining UAE and EAE could exploit the unique advantages of an integrated ultrasound-assisted enzymatic extraction (UAEE) system, which could lead to possible process intensification.

Fucoidans are a potentially high-value sulfated polysaccharides found exclusively in brown seaweeds. They are water-soluble, heterogenous, and mainly consist of sulphated L-fucose units, although they also contain minor amounts of galactose, xylose, glucose, mannose, rhamnose and glucuronic acid [10], [24], [25]. Fucoidans have been reported to exhibit antioxidant [26], [27], anti-diabetic [1], [28], and anti-cancer activities [[3], [13]]. As a result, interest in fucoidan has grown considerably in the last decade due to their potential application in functional foods and for drug development.

This current study aimed to employ a coupled ultrasound-enzymatic extraction method for fucoidan extraction from a South African kelp, *Ecklonia maxima*, and to optimize the extraction conditions through response surface methodology. Additionally, extraction kinetics were monitored to determine the impact of extraction conditions on the rate of fucoidan extraction.

## 2 Materials and methods

### 2.1 Experimental approach

The work set out to evaluate a combined ultrasound assisted enzymatic extraction method. The chosen enzyme was a food-grade, low-cost carbohydrase enzyme complex, *Accellerase® 1500*, due to high activity at mild acidic to neutral pH range (3.9–7) conditions, which is the same region where fucoidan is also soluble. Additionally, the enzyme has multiple activities (endoglucanase, beta-glucosidase, hemi-cellulase and exoglucanase activity) which could facilitate in hydrolyzing the recalcitrant cell wall. A central composite experimental design (CCD) was employed to determine the influence of extraction temperature, ultrasound intensity (UI), enzyme dosage and pH on solubilized yields of total carbohydrates and fucoidan (measured as inorganic sulphates). The experimental data were analysed at endpoint conditions of the experimental runs using response surface methodology (RSM). Extraction kinetics within runs were described and evaluated by fitting the empirical Peleg model.

### 2.2 Chemicals and equipment

Anthrone, D-(+)-glucose, potassium sulphate, barium chloride dihydrate, trichloroacetic acid (TCA), sulphuric acid and hydrochloric acid were obtained from Merck, South Africa. l-cysteine hydrochloride, L-(–)-Fucose and gelatin were obtained from Sigma–Aldrich, South Africa. The enzyme complex, *Accellerase® 1500* was obtained from GENENCOR.

A 24 kHz UP200St ultrasound system with a power output of 200 W and maximum amplitude of 190 µm, fitted with a horn tip transducer of 0.38 cm^2^ in a 12 ml flow cell, was employed as an ultrasound source for the extractions, manufactured by Hielscher, Germany. A stirred, jacketed, 1 l glass bioreactor was used as the extraction vessel and was fitted with a temperature and pH probe. The temperature in the vessel was controlled by circulating heated water (from a temperature-controlled water bath) through the jacket, and pH was automatically controlled by a control system. The bioreactor and control system were manufactured by Glaschem, South Africa.

### 2.3 Algal biomass preparation

Brown seaweed (*Ecklonia maxima*) was harvested from the west coast of South Africa, Cape Town. After collection, the seaweed material was washed with water to remove residual sand and salts. Thereafter, the material was mixed with water at a ratio of 1:1 (mass basis) and then milled through a Comitrol® colloid mill to produce a homogenised slurry, and frozen at −18 °C until used. Before extraction, the samples were defrosted at 4 °C, and analyzed in triplicate at 180 °C to determine moisture content, using a halogen lamp moisture analyzer, a product of Kern Derbs, South Africa.

### 2.4 Extraction methods

**Fig. 1:**
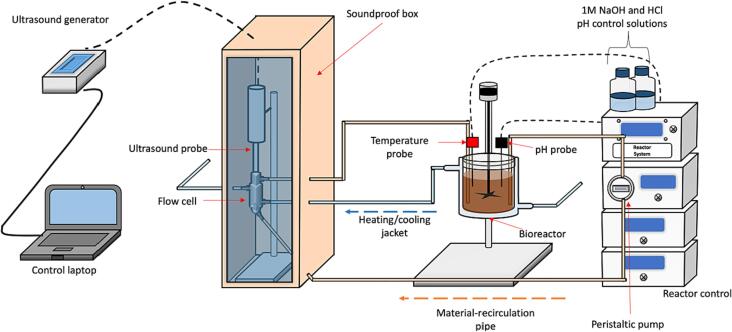
Schematic of experimental set-up for ultrasound-assisted enzymatic extraction (ultrasound-enhanced bioreactor, batch re-circulation). Adapted and redrawn from [29].

Ultrasound-assisted enzymatic extraction was conducted using a stirred and temperature-controlled reactor system, the contents of which were circulated through an ultrasonicated flow cell where an ultrasound probe provided ultrasonication. The schematic representation of the set-up is illustrated Fig. 1 in below:

Extraction was performed by loading an 800 ml volume of seaweed-water mixture that was adjusted to a solid loading of 4 % (w/v) and adjusting to the specific run’s pH and temperature under constant recirculation and mixing via the peristaltic pump and stirrer respectively. Runs were initiated by the addition of enzymes and/or initiating ultrasonication.

The reactor contents were circulated through the system at a constant rate of 92 ml·min^−1^ and sonicated at ultrasound intensity between 0 and 118 W·cm^−2^. The temperature was varied between 40 and 65 °C, enzyme dosage between 0 and 0.05 ml·g^−1^ of biomass, and pH between 4.5 and 6. The extractions were conducted for 240 min, under a constant stirring rate of 200 rpm, and were sampled at 30 min intervals (at times 0, 30, 60, 90, 120, 150, 180, 210 and 240 min). Individual samples constituted 8 ml and were, weighed, and then centrifuged at 5000 rpm for 5 min to separate the liquid hydrolysate (supernatant) and the solids. The supernatant was weighed and then heated at 100 °C for 5 min in a heating block to deactivate the enzymes (the enzymes were denatured, but not removed from the solution). The supernatant was then allowed to cool to room temperature, after which it was split into 2 ml aliquots and stored at −18 °C until analysis (all the analytical assays were carried out on the supernatant, the aqueous samples).

### 2.5 Analytical assays

#### 2.5.1 Concentration of total dissolved solids and solubilized yield

The solubilization of the seaweed material over time was assessed by tracking the dry matter content in the supernatant. Triplicate samples of 500 µl supernatant were analyzed using a halogen lamp moisture analyzer to obtain the dry matter content in the supernatant. The concentration of total dissolved solids at a certain time point, i ([TDS]_i_ in g⋅l^−1^) was obtained from the measurement of the moisture content (M_i_ in % w/v (g/ml) of the sample), and 10 being the conversion factor from % g/ml to g/l calculated using **equation** (1)

(1) $${\left[TDS\right]}_{i}=\left(100-M_{i}\right)\times 10$$

The solubilized yield (in %), which is the fraction of the total dissolved solids in the hydrolysate to the initial total solids in the medium ([TS]_o_) in g⋅l^−1^ as was calculated by **equation** (2)

(2) $$Solubilizedyield\left(\%\right)=\frac{{\left[TDS\right]}_{i}-{\left[TS\right]}_{0}}{{\left[TS\right]}_{0}}\times 100$$

#### 2.5.2 Total carbohydrates

The total carbohydrate content of the extract was measured by the anthrone assay with some modifications [30]. The anthrone reagent was prepared by adding 200 mg of anthrone powder to 100 ml of ice-cold 95 %(w/v) sulphuric acid, thoroughly mixed and kept at 4 °C until required. To analyze samples, 200 µl of extract was hydrolyzed in 1.8 ml of 4 N HCl at 105 °C for 2 h to hydrolyze carbohydrates into monosaccharides, followed by cooling in an ice-cold bath. After cooling, 100 µl of the hydrolyzed extract was diluted by adding 400 µl of deionized water, and vortexed for 1 min to mix. The diluted sample (500 µl) and anthrone reagent (1 ml) were mixed and incubated at 90 °C for 10 min, followed by cooling in an ice bath. Absorbance was measured at 630 nm using a microplate reader ELx800 (BioTek Instruments, United States), and values were determined against a glucose standard curve in the range of 0–0.1 mg⋅ml^−1^ and expressed as mg glucose equivalents. The results were expressed as yield of glucose equivalents (mg) per gram of initial seaweed raw material loaded into solution.

#### 2.5.3 Inorganic sulphates

The sulphate content of the extract was measured by the barium chloride-gelatin method with some modifications [31]. The barium chloride-gelatin reagent was prepared by mixing 0.5 g gelatin powder into 100 ml of water at temperatures between 60 and 70 °C. The gelatin solution was stored at 4 °C overnight, before adding 0.5 g of barium chloride. 3 % (w/v). A trichloroacetic acid (TCA) solution was prepared by adding 3 g of TCA to 100 ml of deionized water.

For sample analysis, 200 µl of crude extract was diluted with 600 µl of deionized water and vortexed for 1 min. Of this diluted extract, 80 µl was added to 400 µl of barium chloride-gelatin reagent and 1.52 ml of TCA, mixed and incubated at 25 °C for 20 min. Absorbance was measured at 490 nm using a microplate reader. The measurements were quantified using K_2_SO_4_ as standard between 0 and 1 mg SO_4_^2-^⋅ml^−1^, and results were used to determine the yield of sulphates (mg sulphates per g of initial seaweed raw material loaded into solution).

#### 2.5.4 Fucose

The fucose content of the extract was measured by Dische’s assay [32]. A 3 % (w/v) solution of L-cysteine hydrochloride was prepared by adding 3 g of L-cysteine hydrochloride to 100 ml of deionized water. To analyze samples, 200 µl of crude extract was mixed with a 900 µl solution of 6:1 (v/v) 95 % sulphuric acid, and incubated for 3 min at 25 °C, followed by incubation at 90 °C for 3 min. Immediately after incubation, the samples are cooled in an ice bath. After cooling, 50 µl of 3 % (w/v) L-cysteine hydrochloride solution was added to the sample solution and mixed. These samples were incubated at 25 °C in the dark for 30 min. Absorbances were measured at 396 nm and 430 nm using a UV/Vis spectrophotometer, model: AE-S60-4U (manufactured by A & E laboratories, United Kingdom) and effective absorbance was taken as the difference between absorbances at 396 nm and 430 nm. The measurements were quantified using a fucose standard curve (0–0.1 mg⋅ml^−1^). the results were expressed as the yield of fucose (mg fucose per g of initial seaweed material loaded in solution).

### 2.6 Experimental design

A central composite design (CCD) with four factors and five levels was used to investigate and optimize process variables (temperature (40–65 °C), ultrasound intensity (0–118 W⋅cm^−2^), enzyme dosage (0–0.05 ml⋅g^−1^ biomass) and pH (4.5–6)), with solubilized yield, total carbohydrates and inorganic sulphates yields as response variables. Inorganic sulphates were employed as indicators of fucoidan as done previously [14], as sulphates are functional groups in the fucoidan molecule. The ranges for temperature and pH were chosen based on the enzyme cocktail’s operating region, and the range for enzyme dosage was based on the supplier specification sheet and literature [33], [34], [35]. The range for ultrasound intensity was based on the equipment's capabilities. The experimental design is illustrated in Table 1, and comprised of a standard CCD and 6 center point replicates, with α values of ± 2.

**Table 1:** Central composite design matrix for four factors at five level settings used for optimization.

Coded Level	Parameters symbols -Original values			
	X_1_ – Temperature °C	X_2_ – UI W·cm^−2^	X_3_ - Enzyme dosage ml·g^−1^_biomass_	X_4_ - pH
α	65.00	118.33	0.0500	6.00
1	58.75	88.75	0.0375	5.63
0	52.50	59.16	0.0250	5.25
−1	46.25	29.58	0.0125	4.88
-α	40.00	0	0	4.50

Regression was employed to determine optimal values for operating conditions, by fitting experimental data to a quadratic regression model (**Equation** (3)) and determining regression coefficients. Desirability analysis and response surface methodology (RSM) aided in optimization.

(3) $$Y_{o}=\beta_{o}+\sum_{i=1}^{4}\beta_{i}X_{i}+\sum_{i=1}^{4}\beta_{\mathrm{ii}}{X_{i}}^{2}+\sum_{i=1}^{4}\sum_{j=i+1}^{4}\beta_{\mathrm{ij}}X_{i}X_{j}$$

Where $Y_{o}$ represents the response variable, and *β_o_, β_i_, β_ii_* and *β_ij_* represent the regression coefficients for intercept, linear, quadratic and interaction respectively; *X_i_* and *X_j_* represent two independent variables, where i ≠ j. Model regression, RSM and desirability were all carried out using Statistica (Version 13.3, TIBCO Software, USA).

### 2.7 Kinetic modelling

The model proposed by Peleg was adapted and used for the description of fucoidan extraction kinetics by the UAEE process. Although the model was initially used to describe sorption kinetics, it has been used successfully to describe extraction kinetics of various extracts from plant material [36], [37], [38]. The model given by **Equation** (4) was fitted to concentration–time series data of total dissolved solids, total carbohydrates, and inorganic sulphates.

(4) $$C\left(t\right)=C_{o}+\frac{t}{K_{1}+K_{2}t}$$

Where $C\left(t\right)$ is the concentration or yield of TDS (g·l^−1^) or total carbohydrates (mg·g^−1^) or inorganic sulphates (mg·g^−1^) at time t, $C_{o}$ is the concentration or yield of TDS (g·l^−1^) or total carbohydrates (mg·g^−1^) or inorganic sulphates (mg·g^−1^) at time t = 0, $K_{1}$ (min·g·mg^−1^ or min·l·g^−1^) is the Peleg’s rate constant, $K_{2}$ (g·mg^−1^ or l·g^−1^) is the Peleg’s capacity constant and t is the extraction time. Peleg’s rate constant is related to the extraction the rate ($B_{o}$) at the beginning of the extraction process, and Peleg’s capacity constant is related to the equilibrium concentration of total dissolved solids or total carbohydrates or inorganic sulphates ($C_{e})$ given by **Equation** (5) and **Equation** (6) respectively.

(5) $$\frac{\mathrm{dC}(t)}{\mathrm{dt}}{|}_{t=0}=B_{0}=\frac{1}{K_{1}}$$

(6) $${C(t)}_{t\to \infty }=C_{e}=C_{0}+\frac{1}{K_{2}}$$

### 2.8 Statistical analysis

Unless otherwise stated, results are expressed as means ± standard deviations. Statistica (version 13.3, TIBCO, USA) was used for all statistical analysis, including desirability analysis and response surface methodology. Analysis of variance (ANOVA) was used to analyze data obtained at the end of extractions (at 240 min), to assess the influence of the operating variables on the extraction, and their possible interaction. Third and higher order interactions were omitted from RSM due to difficulty in connecting physical meanings to these interactions. Differences between means were taken as statistically significant at a significance level of 95 % (p < 0.05). Tukey’s HSD test was performed as post-hoc test to determine which means differed significantly.

Peleg models were fitted to experimental data using non-linear regression, using the method of least squares. Regression was carried out in Microsoft Office 16 Excel, using the solver function. The models’ adequacy and goodness of fit between experimental data and predicted data were determined based on the coefficient of determination (R^2^) and root mean square error (RMSE). Star point runs that denote the extremes of the CCD, and centre point experimental runs (-α, 0, α) of the CCD were used for kinetic modelling and analysis.

## 3 Results

### 3.1 Optimization of parameters for extracting polysaccharides through UAEE

The experimental results of the four independent experimental factors (temperature, ultrasound intensity, enzyme dosage and pH) are presented in Table 2. The results of the ANOVA and the regression coefficients for the quadratic regression equation are summarized in Table 3**.** The regression equations are presented by Equations 9–11:

(7) $$C_{\mathrm{TDS},240}=32.46+{0.87X}_{1}+{1.26X}_{2}+{0.92X}_{3}-{0.25X}_{4}-0.31{X_{1}}^{2}-0.44{X_{2}}^{2}-{{0.37X}_{3}}^{2}+0.10{X_{4}}^{2}-0.99X_{1}X_{2}-0.45X_{1}X_{3}+0.05X_{1}X_{4}-{0.76X}_{2}X_{3}+{0.16X}_{2}X_{4}+0.43X_{3}X_{4}$$

(8) $$Y_{\mathrm{TC},240}=75.32+{5.50X}_{1}+{6.30X}_{2}+{7.53X}_{3}-{0.06X}_{4}-10.73{X_{1}}^{2}-7.50{X_{2}}^{2}+1.23{X_{3}}^{2}-6.22{X_{4}}^{2}+4.29X_{1}X_{2}+3.75X_{1}X_{3}-1.97X_{1}X_{4}+{7.69X}_{2}X_{3}+{9.91X}_{2}X_{4}-0.95X_{3}X_{4}$$

(9) $$Y_{\mathrm{IS},240}=26.20+{1.09X}_{1}+{1.87X}_{2}+{0.43X}_{3}+{0.51X}_{4}-3.81{X_{1}}^{2}-2.16{X_{2}}^{2}-{{1.92X}_{3}}^{2}-1.88{X_{4}}^{2}-1.14X_{1}X_{2}-0.30X_{1}X_{3}+1.55X_{1}X_{4}-{1.52X}_{2}X_{3}+{2.90X}_{2}X_{4}-0.28X_{3}X_{4}$$

$X_{1},X_{2},X_{3}andX_{4}$ represent temperature, ultrasound intensity, enzyme dosage and pH respectively. For the concentration of total dissolved solids (C_TDS,_ g⋅l^−1^), at 240 min, the model R^2^ was 0.44, and the *p*-value for the lack of fit was 0.12 The model *p*-value > 0.05 indicates that the lack of fit is not significant, however the R^2^ value was low at 0.44, indicating that the majority of the variation in the model was not explained by the experimental factors. Data for carbohydrate (Y_TC,_ mg glucose⋅g^−1^) and sulphate yield (Y_IS_, mg SO_4_^2-^⋅g^−1^ at the end of extractions were also regressed to fit quadratic models. For both the fitted regression models the lack of fit was not significant (p > 0.05), and the R^2^ values were 0.74 and 0.84 for total carbohydrate and inorganic sulphates yields respectively.

**Table 2:** Complete central composite design matrix and the response values for total dissolved solids, total carbohydrates and inorganic sulphates yields used for optimization.

**St. Order**	**Temperature (°C)**	**UI (W**⋅**cm^-^^2^)**	Enzyme dosage **(ml**⋅**g^-1^)**	**pH**	**Total dissolved solids (g**⋅**l^−1^)**	**Total carbohydrate (mg glucose equivalent**⋅**g^−1^)**	**Inorganic sulphates (mg SO_4_^2-^**⋅**g^−1^)**
1	46.25	29.58	0.0125	4.88	31.40	59.46	18.46
2	46.25	29.58	0.0125	5.63	29.08	59.93	16.80
3	46.25	29.58	0.0375	4.88	30.67	61.08	22.29
4	46.25	29.58	0.0375	5.63	31.07	44.53	22.12
5	46.25	88.75	0.0125	4.88	30.73	46.29	24.47
6	46.25	88.75	0.0125	5.63	32.50	59.36	22.18
7	46.25	88.75	0.0375	4.88	30.27	54.05	20.26
8	46.25	88.75	0.0375	5.63	33.90	67.31	21.72
9	58.75	29.58	0.0125	4.88	30.27	67.38	21.49
10	58.75	29.58	0.0125	5.63	33.77	56.52	22.50
11	58.75	29.58	0.0375	4.88	34.63	67.06	19.86
12	58.75	29.58	0.0375	5.63	31.57	56.74	24.34
13	58.75	88.75	0.0125	4.88	32.43	61.29	20.43
14	58.75	88.75	0.0125	5.63	33.10	64.77	22.64
15	58.75	88.75	0.0375	4.88	31.95	72.90	21.43
16	58.75	88.75	0.0375	5.63	32.35	85.07	23.50
17	40	59.17	0.025	5.25	32.93	61.77	16.87
18	65	59.17	0.025	5.25	31.73	54.88	19.45
19	52.5	0.00	0.025	5.25	30.08	55.48	18.03
20	52.5	118.33	0.025	5.25	34.10	74.11	24.87
21	52.5	59.17	0	5.25	30.83	68.11	22.26
22	52.5	59.17	0.05	5.25	33.63	96.41	21.59
23	52.5	59.17	0.025	4.50	34.57	69.08	22.26
24	52.5	59.17	0.025	6.00	31.77	65.64	21.78
25	52.5	59.17	0.025	5.25	32.60	84.79	27.48
26	52.5	59.17	0.025	5.25	33.20	70.25	26.43
27	52.5	59.17	0.025	5.25	34.25	77.78	24.30
28	52.5	59.17	0.025	5.25	31.13	65.01	25.48
29	52.5	59.17	0.025	5.25	31.73	76.95	26.54
30	52.5	59.17	0.025	5.25	31.87	77.16	26.97

Experimental design provided in standard order. St. Order represent standard order. Response values are means of three independent measurements.

**Table 3:** Analysis of variance and regression coefficients for second order polynomial models in terms of actual variables.

	**Total dissolved solids yield**			**Total carbohydrate yield**			**Inorganic sulphates yield**		
**Coefficient**	**Effect estimate**	**t-Ratio**	**P-value**	**Effect estimate**	**t-Ratio**	**P-value**	**Effect estimate**	**t-Ratio**	**P-value**
Intercept									
β_o_	32.46	70.22	0.00 ***	75.32	26.98	0.00 ***	26.20	56.12	0.00 ***
Linear									
β_1_	0.87	1.87	0.11	5.50	1.97	0.10	1.09	2.33	0.06
β_2_	1.26	2.73	0.04 *	6.30	2.26	0.07	1.87	4.01	0.01 *
β_3_	0.92	2.00	0.10	7.53	2.70	0.04 *	0.43	0.93	0.3950
β_4_	−0.25	−0.53	0.61	−0.18	−0.06	0.9511	0.51	1.10	0.3223
Quadratic									
β_11_	−0.31	−0.73	0.50	−10.73	−4.11	0.01**	−3.81	−8.72	0.00 ***
β_22_	−0.44	−1.01	0.36	−7.50	−2.87	0.03 *	−2.16	−4.95	0.00 **
β_33_	−0.37	−0.85	0.43	1.23	0.47	0.65	−1.92	−4.40	0.01 **
β_44_	0.10	0.24	0.82	−6.22	−2.38	0.06	−1.88	−4.30	0.01 **
Interaction									
β_12_	−0.99	−1.75	0.14	4.29	1.25	0.26	−1.14	−2.00	0.1019
β_13_	−0.45	−0.79	0.46	3.74	1.09	0.32	−0.30	−0.53	0.6208
β_14_	0.05	0.08	0.93	−1.97	−0.58	0.58	1.55	2.72	0.04 *
β_23_	−0.76	−1.34	0.23	7.69	2.25	0.07	−1.52	−2.66	0.04 *
β_24_	0.16	0.88	1.28	9.91	2.90	0.03 *	−0.03	−0.05	0.9652
β_34_	0.43	0.69	0.01 *	−0.95	−0.28	0.79	1.07	1.87	0.1199
									
Model	df (SS)		P-value	df (SS)		P-value	df (SS)		P-value
Lack of fit	10 (38.99)		0.12	10 (735.31)		0.32	10 (30.75)		0.18
R^2^	0.44			0.74			0.84		

βo is the intercept constant, β_i_, β_ii_ and β_ij_ are the linear quadratic and interaction coefficient for the second order polynomial equation. df is degree of freedom. Subscripts 1,2,3 and 4 represent temperature, ultrasound intensity, enzyme dosage and pH respectively. SS is sum of squares, and R^2^ is the coefficient of determination. *, **, *** indicates significance at P-value < 0.05, 0.01 and 0.001 respectively.

### 3.2 Impact of extraction parameters on yield

#### 3.2.1 Total dissolved solids concentration (solubilized yield)

**Fig. 2:**
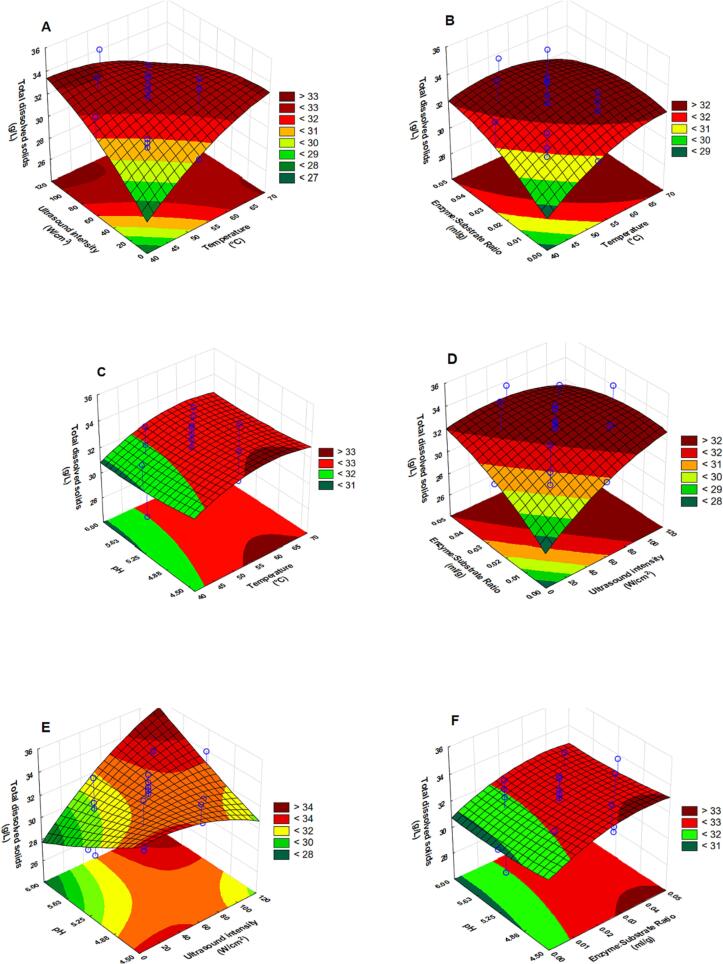
Response surface plots showing interactive effects of temperature and (ultrasound intensity (A), enzyme dosage (B), pH (C)), of ultrasound intensity and (enzyme dosage (D), pH (E)), of enzyme dosage (F) on the endpoint concentration of total dissolved solids.

The influence of extraction parameters on the endpoint yield of total dissolved solids was evaluated through ANOVA (summarized in and Table 3) and by the graphical approach of RSM (illustrated by the response surfaces in Fig. 2). ANOVA at the endpoint revealed that the linear effect of ultrasound intensity (X_2_) and interaction effect between enzyme dosage and pH (X_3_X_4_) were significant.

Fig. 2 (A) shows that the concentration of total dissolved solids increases with increasing ultrasound intensity. Increasing ultrasound intensity from 0 to 118 W⋅cm^−2^ resulted in an increase of total dissolved solids concentration from 27 g⋅l^−1^ to approximately 33 g⋅l^−1^. Fig. 2 (B, D and F) all highlight that increasing enzyme dosage increases the concentration of total dissolved solids. The impact of pH is seen to be minimal, although, the interaction term suggest that a combination of higher pH and high enzyme dosage would lead to higher concentration of total dissolved solids. Therefore, the solubilized yield would be maximized at high enzyme dosage and higher pH’s of the experimental domain (at constant ultrasound intensity and temperature).

#### 3.2.2 Total carbohydrates yield

**Fig. 3:**
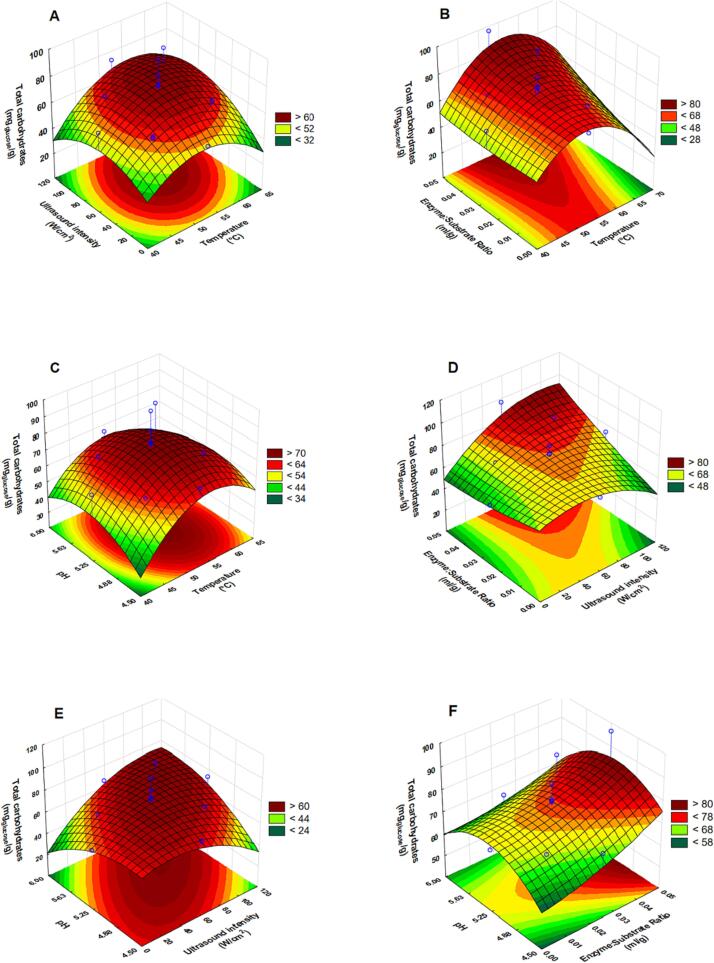
Response surface plots showing interactive effects of temperature and (ultrasound intensity (A), enzyme dosage (B), pH (C)), of ultrasound intensity and (enzyme dosage (D), pH (E)), of enzyme dosage (F) on the endpoint yield of total carbohydrates.

The results of the endpoint yield of total carbohydrates across the experimental domain are summarized in Table 2. The endpoint yield of total carbohydrates ranged from 44 mg⋅g^−1^ (at 46.25 °C, 29.58 W·cm^−2^, enzyme dosage 0.0375 ml·g^−1^, pH 5.63) to 96 mg·g^−1^ (at 52.5 °C, 59.17 W·cm^−2^, enzyme dosage 0.05 ml·g^−1^, pH 5.63). ANOVA results and the fitted empirical quadratic model are given in and **Equation** (8) respectively. The influence of extraction parameters on the yield is illustrated in Fig. 3 (A–F). ANOVA results revealed that the linear effect of enzyme dosage (X_3_), quadratic effects of temperature (X_1_^2^) and ultrasound intensity (X_2_^2^) and the interaction effect between ultrasound intensity and pH (X_2_X_4_) were significant.

Fig. 3**(A)** shows that, at constant enzyme dosage and pH, the yield of total carbohydrates increased with increasing temperature from 40 to about 55 ℃, after which, a further increase in temperature resulted in a decrease in the yield of total carbohydrates. Similarly, for an increasing ultrasound intensity, the yield increased between 0 and 60 W·cm^−2^, after which a decrease in yield was observed (Fig. 3(A, D, E)). Noteworthy, at a high enzyme dosage (0.05 ml·g^−1^), the decrease in yield with increasing ultrasound intensity is not prominent (Fig. 3 (D)). Fig. 3**(E)** shows that a combination of higher pH (around 6) with high ultrasound intensity resulted in high yield of total carbohydrates.

#### 3.2.3 Inorganic sulphates

The endpoint yields of inorganic sulphates across the experimental domain are summarized in Table 2. The yield ranged from 16.8 mg SO_4_^2-^⋅g^−1^ (46.25 °C, 29.58 W·cm^−2^, enzyme dosage 0.0125 ml·g^−1^, pH 5.63) to 27.48 mg SO_4_^2-^·g^−1^ (52.5 °C, 99.17 W·cm^−2^, enzyme dosage 0.025 ml·g^−1^, pH 5.25). ANOVA results (Table 3) revealed the linear effect of ultrasound intensity (X_2_) to be significant. Additionally, the quadratic effects of all the independent variables were found to be significant. The results also revealed that the interaction effects between temperature and pH (X_1_X_2_) as well as between ultrasound intensity and enzyme dosage (X_2_X_3_) were significant.

**Fig. 4:**
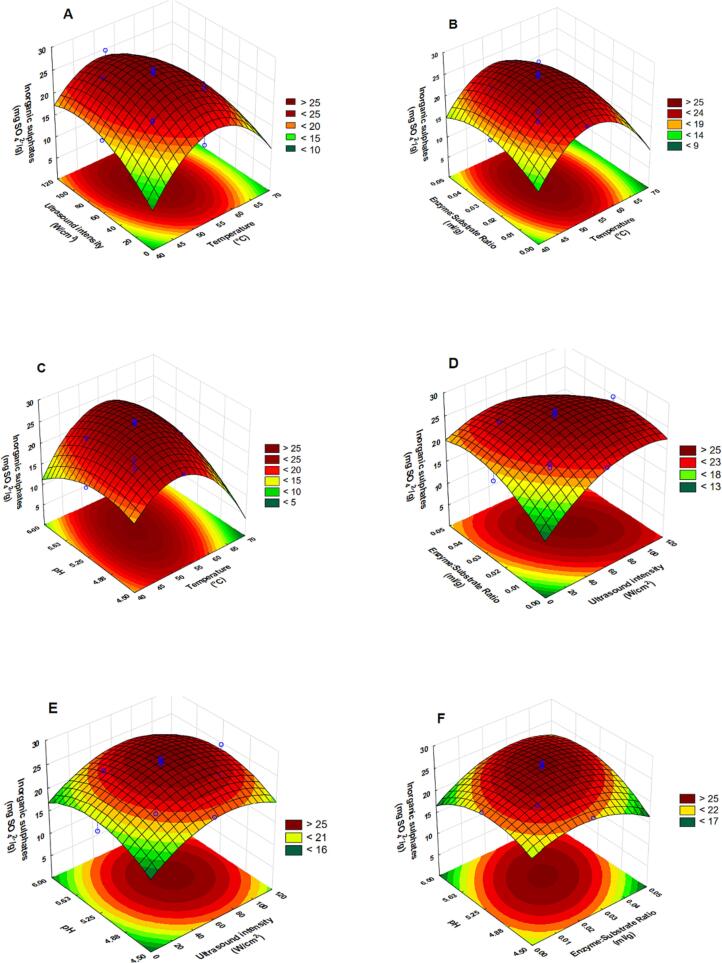
Response surface plots showing interactive effects of temperature and (ultrasound intensity (A), enzyme dosage (B), pH (C)), of ultrasound intensity and (enzyme dosage (D), pH (E)), of enzyme dosage (F) on the endpoint yield of inorganic sulphates.

All the coefficients of the quadratic effects were found to be negative, which suggests that curvature exists for all the effects within the experimental domain. It is also implied that the factors' influence on the yield is characterized by an increase with an increasing change in the factors, until a threshold, after which the yield will start decreasing with a further increase in the factor. Inorganic sulphate yield increased with an increasing temperature between 40 and 55 °C (Fig. 4(A-C)), which is similar to the observation of the effect on the yield of total carbohydrates. The yield increased between 0–80 W⋅cm^−2^ (Fig. 4(A, D and E), 0–0.03 ml⋅g^−1^ (Fig. 4**(F)**) and 4.5–5.5 ((C and E) for ultrasound intensity, enzyme dosage and pH respectively. Fig. 4**(C)** shows that the yield of inorganic sulphates is high at a combination of elevated temperatures and high pH.

### 3.3 Optimal conditions of UAEE and model validation

A set of optimum conditions for extracting total carbohydrates and inorganic sulphates were determined using desirability analysis (summarized in Table 4). Multi-variables optimization by desirability has been used and described recently by [39]. The optimal conditions for simultaneous extraction of total carbohydrates and inorganic sulphates were at 52.5 °C, ultrasound intensity of 59.17 W·cm^−2^, enzyme dosage of 0.0375 ml·g^−1^ and pH of 5.25, with predicted yields of 79.19 mg glucose equivalents ·g^−1^ and 25.46 mg SO_4_^2-^·g^−1^ respectively.

**Table 4:** Optimum conditions for ultrasound-assisted enzymatic extraction (UAEE) of fucoidan from *Ecklonia maxima.*

**Desirability profile**	**Optimum conditions**	**Predicted yield (at 240 min)**	**Validation**
Simultaneous desirability on total carbohydrates and inorganic sulphates yield.	X1 - Temperature (52.5 °C)X2 – Ultrasound intensity (59.17 W·cm^−2^)X3 – Enzyme dosage (0.0375 ml·g^−1^)X4 - pH (5.25)	Total carbohydrates: 79. 19 mg glucose·g^−1^ 25.46 mg SO_4_^2-^·g^−1^	N/A
Inorganic sulphates yield based on the reduced model.	X1 - Temperature (58.75 °C)X2 – Ultrasound intensity (88.75 W⋅cm^−^^2^) X3 – Enzyme dosage (0 ml⋅g^−1^)X4 - pH (6)	Inorganic sulphates: 24.89 mg SO_4_^2-^·g^−1^	Inorganic sulphates: 23.74 ± 1.56 mg SO_4_^2-^·g^−1^

The main aim of this study was to optimize fucoidan yield, based on inorganic sulphates yield. Desirability function was used on the reduced model of the model provided by **equation** (9). The reduced model was based on sequentially omitting the insignificant terms in descending order of *p-values*. Significant variables and those which are slightly insignificant (0 < *p-value* < 1) were included in the reduced model, resulting in R^2^ = 0.77 and adjusted R^2^ = 0.71. The reduced model of inorganic sulphates is provided by **equation** (10). The optimized level of process parameters indicated that 58.75 °C, ultrasound intensity of 88.75 W·cm^−2^, enzyme dosage of 0 ml·g^−1^ and pH 6 give inorganic sulphates yield of 24.89 mg SO_4_^2-^·g^−1^ with a desirability value of 0.76.

(10) $$Y_{\mathrm{IS},240*}=26.20+{1.09X}_{1}+{1.87X}_{2}-3.81{X_{1}}^{2}-2.16{X_{2}}^{2}-{{1.92X}_{3}}^{2}-1.88{X_{4}}^{2}+1.55X_{1}X_{4}-{1.52X}_{2}X_{3}$$

**Fig. 5:**
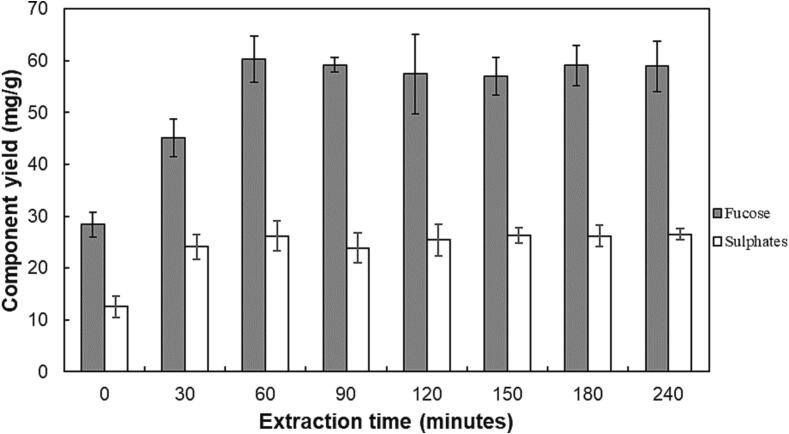
Validation experimental results of fucose and inorganic sulphates at optimum conditions. Temperature = 58.75 °C, ultrasound intensity = 88.75 W⋅cm^−2^, enzyme dosage = 0 ml⋅g^−1^, pH = 6.

A validation run was carried out under the predicted optimal extraction conditions using the reduced model, and compared to the predicted equilibrium yield. Under the optimum conditions, the inorganic sulphate yield was found to be 23.74 ± 1.52 mg SO_4_^2-^·g^−1^, compared to the predicted value of 24.89 mg SO_4_^2-^·g^−1^. To verify the presence of fucoidan, fucose yields were also measured and are shown, along with sulphate yields, in Fig. 5. There was a significant positive correlation between the extraction of fucose and inorganic sulphates, as shown by the high Pearson correlation coefficient of 0.91, with a corresponding p-value < 0.05. The ratio of inorganic sulphates: fucose ratio was 0.45 ± 0.03 (average ratio for all time points). reported a similar inorganic sulphate: fucose ratio of 0.57 for fucoidan extracted by hot water from *Ecklonia maxima*. The following results suggest the extraction of sulphated fucose (fucoidan). The sulphate content (% w/w) of the fucoidan was found to be 31.05 ± 1.60 %. It has been reported that the fucoidan sulphate varies between 20 and 30 % (w/w) of the fucoidan compound [40], and the current fucoidan lies on the high end of the range.

### 3.4 Kinetic modelling

The results of fitting Peleg’s model to selected extractions are summarized in Table 5. The table details the kinetic rate constant (*K_1_*) and the capacity constant (*K_2_*) obtained during curve fitting, the initial extraction rates (*B_o_*) and the predicted equilibrium yields (*C_e_*), which are calculated from *K_1_* and *K_2_*, and the R^2^and the RMSE, which describes how well the models fit the data. In general, the models had relatively high R^2^ values, which ranged between 0.70 and 0.98, 0.89–0.98 and 0.75–0.99 for total dissolved solids, total carbohydrates, and inorganic sulphates respectively.

**Table 5:** Effects of extraction temperature, ultrasound intensity, enzyme dosage and pH on the Peleg’s kinetics parameters for total dissolved solids, total carbohydrates, and inorganic sulphates.

		Total dissolved solids yield							Total carbohydrates yield							Inorganic sulphates yield						
Factor	**Level**	***K_1_***	***K_2_***	***C_o_*** (g·l^−1^)	***B_o_*** (g·l^−1^·min^−1^)	***C_e_*** (g·l^−1^)	***R^2^***	***RMSE***	***K_1_***	***K_2_***	***C_o_***(mg⋅g^−1^)	***B_o_***(mg·g^−1^·min^−1^)	***C_e_***(mg·g^−1^)	***R^2^***	***RMSE***	***K_1_***	***K_2_***	***C_o_***(mg.g^−1^)	***B_o_***(mg·g^−1^·min^−1^)	***C_e_*** (mg·g^−1^)	***R^2^***	***RMSE***
Temperature (°C)	40.00	8.60	0.09	25.20	0.06	36.59	0.95	0.60	1.35	0.04	44.55	0.74	67.48	0.92	1.82	0.69	0.12	12.93	1.44	21.30	0.79	1.34
	52.50	16.52	0.11	26.96	0.12	35.86	0.98	0.27	0.55	0.03	44.86	1.83	78.19	0.99	1.36	0.67	0.09	15.44	1.50	26.40	0.98	0.73
	65.00	5.43	0.13	25.70	0.18	33.68	0.95	0.46	0.88	0.03	41.27	1.14	74.60	0.81	3.30	1.00	0.13	14.89	1.00	22.42	0.70	1.72
Ultrasound intensity (W/cm^2^)	0.00	15.43	0.17	26.64	0.06	32.36	0.89	0.45	1.41	0.06	41.18	0.71	57.81	0.75	2.74	5.84	0.10	10.51	0.17	20.67	0.87	1.00
	59.16	14.32	0.13	26.66	0.07	34.40	0.95	0.42	0.55	0.03	44.86	1.83	78.19	0.99	1.36	0.67	0.09	15.44	1.50	26.40	0.99	0.73
	118.33	4.06	0.11	25.71	0.25	34.45	0.93	0.83	3.04	0.02	43.24	0.33	96.68	0.99	1.07	2.77	0.07	11.20	0.36	24.97	0.92	1.55
Enzyme dosage (ml.g^−1^)	0.000	4.68	0.20	26.73	0.21	31.72	0.84	0.61	1.41	0.04	40.80	0.71	68.26	0.88	2.70	1.33	0.08	12.15	0.75	25.45	0.92	1.18
	0.025	14.32	0.13	26.66	0.07	34.40	0.94	0.42	0.55	0.03	44.86	1.83	78.19	0.99	1.36	0.67	0.09	15.44	1.50	26.40	0.97	0.73
	0.050	2.54	0.11	26.07	0.39	35.13	0.96	0.65	1.28	0.02	45.53	0.78	103.39	0.96	4.58	0.63	0.08	12.74	1.60	25.63	0.83	1.89
pH	4.50	14.48	0.09	27.19	0.07	37.83	0.92	0.62	0.95	0.04	44.30	1.05	71.63	0.93	2.12	3.48	0.09	13.16	0.29	24.66	0.81	1.47
	5.25	14.32	0.13	26.66	0.07	34.40	0.95	0.42	0.55	0.03	44.86	1.83	78.19	0.99	1.36	0.67	0.09	15.44	1.50	26.40	0.98	0.73
	6.00	8.95	0.19	26.77	0.11	32.00	0.91	0.57	0.92	0.05	44.64	1.08	66.12	0.87	2.61	11.31	0.11	13.46	0.09	22.50	0.90	1.15

The time series data used for kinetic analysis corresponds to the α (2, −2) settings for each factor and the average of the replicated centre (0) runs. These runs correspond to St. order 17 – 24 and the mean of run 25–30 as the replicates. When one factor is changed, all the other factors are maintained at 0 level setting. K_1_ represents the Peleg’s kinetic rate constant, and K_2_ represents the capacity constant. C_o_, B_o,_ and C_e_ represents model predicted yield at t = 0 min, initial extraction rate and the predicted equilibrium yield at each condition respectively.

**Fig. 6:**
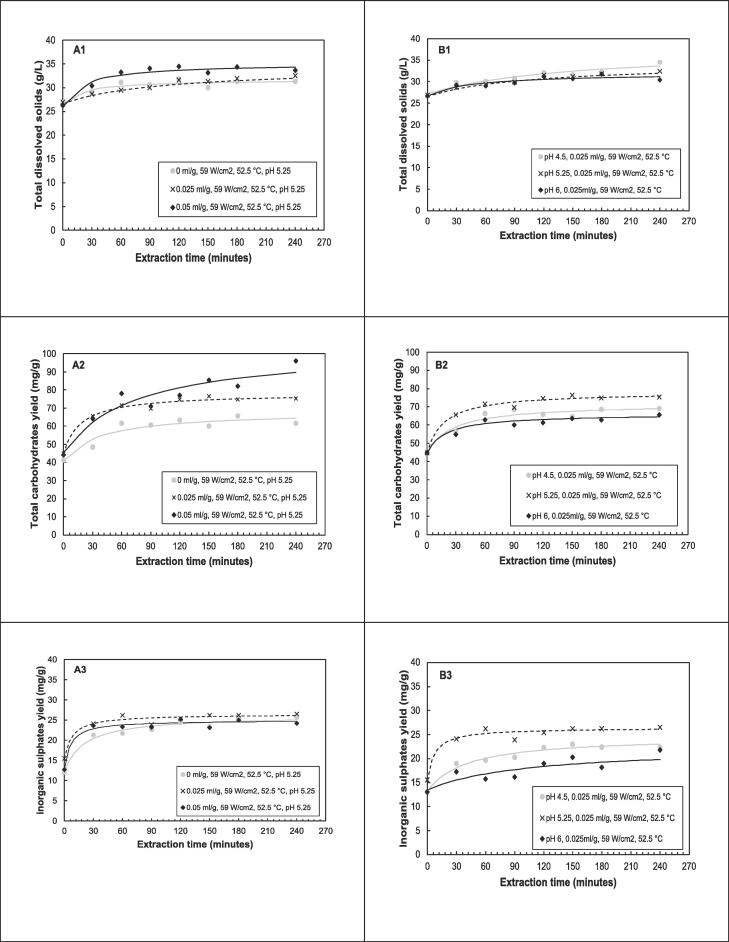
The influence of (A) enzyme dosage and (B) pH on yield of total dissolved solids, total carbohydrates, and inorganic sulphates (symbols – represents experimental data; lines – represents Peleg’s model kinetic curves).

The time-series experimental data and the fitted models are shown in and Fig. 6. It is evident from the figures that irrespective of the extraction conditions, the yields of total dissolved solids, total carbohydrates and inorganic sulphates increased with increasing extraction time, and that extraction profiles are characterized by an initial step with high extraction rates, followed by a slower rate as equilibrium is approached. These profiles are similar to sorption kinetics initially described by [41].

#### 3.4.1 Effect of temperature

**Fig. 7:**
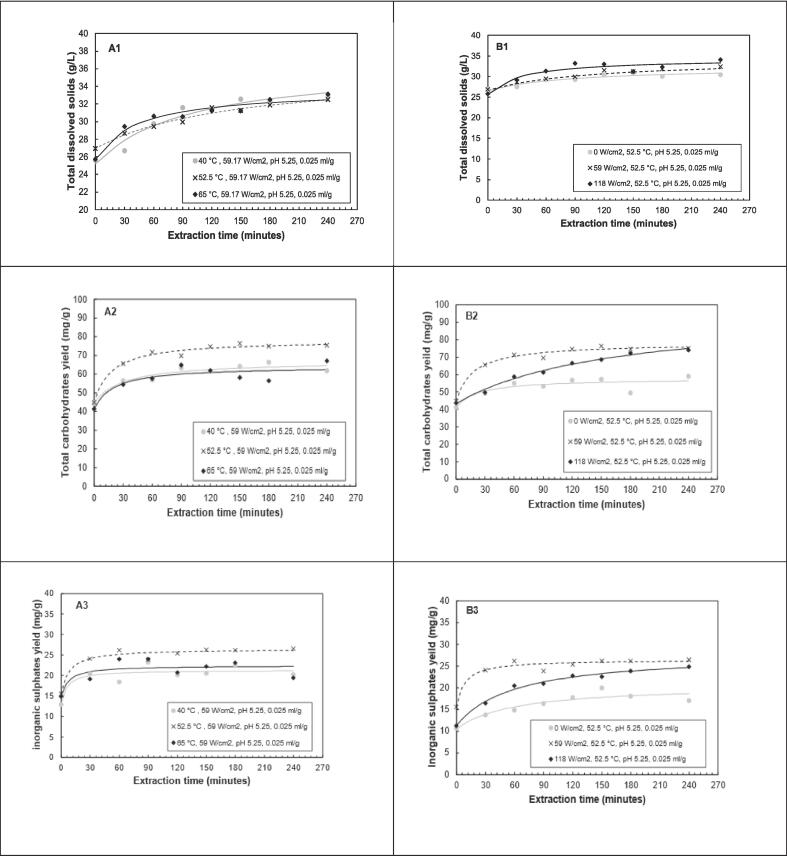
The influence of (A) temperature and (B) Ultrasound intensity on the yield of total dissolved solids, total carbohydrates, and inorganic sulphates (Symbols – represent experimental data; lines – represent Peleg’s model approximation curves).

The influence of extraction temperature on the kinetics of total dissolved solids, total carbohydrates and inorganic sulphates yield was investigated at temperatures of 40, 52.5 and 65 °C while the other factors were at center point values of the CCD. The results in Table 4 reveal that the initial extraction rate (*B_o_*) increased with increased temperature from 40 to 65 °C for total dissolved solids and total carbohydrates. The initial extraction rate increased from 0.06 to 0.18 g·l^−1^·min^−1^ for total dissolved solids and from 0.74 to 1.14 mg·g^−1^·min^−1^ for total carbohydrates yield. The initial extraction rate for inorganic sulphates at 45 and 52.5 °C (1.44 and 1.50 mg·g^−1^·min^−1^) were higher compared to 65 °C (1.00 mg·g^−1^·min^−1^). The extent of extractions (equilibrium yield) was higher at mid ranges of temperatures for total carbohydrates and inorganic sulphates. However, for total dissolved solids, 40 °C and 52.5 °C had comparable equilibrium yields of 36.59 and 35.86 g·L^−1^ respectively (Fig. 7(A_2_–A_3_)).

#### 3.4.2 Effect of ultrasound intensity

The results on the influence of ultrasound intensity on the kinetics **(**Table 5) reveal that the initial extraction rate and the predicted equilibrium yields were higher for experimental runs when ultrasound (59 and 118 W·cm^−2^) is applied compared to when it is not (0 W·cm^−2^). The highest initial extraction rates were at 59 W·cm^2^ for total carbohydrates (1.83 mg·g^−1^·min^−1^) and inorganic sulphates (1.50 mg·g^−1^·min^−1^). The predicted equilibrium yields were highest at high ultrasound intensity for total dissolved solids (34.45 g·l^−1^) and total carbohydrates (96.68 mg·g^−1^). The evolution of the yields with time as influenced by ultrasound intensity are shown in Fig. 7(B_1_-B_2_).

#### 3.4.3 Effect of enzyme dosage

Fig. 6(A_1_-A_3_) summarize the results of the influence of enzyme dosage on kinetics. The results reveal that the highest predicted equilibrium yields for total dissolved solids (35.13 g·l^−1^) and total carbohydrates are at the highest enzyme dosage investigated (0.05 ml·g^−1^). The influence of enzyme dosage on the predicted equilibrium yield of inorganic sulphates was not clear ((A_3_)), as the yields were similar. The effect on the initial extraction rate shows that the highest rate was at 0.025 ml·g^−1^ for both total carbohydrates and inorganic sulphates. For total dissolved solids, high initial extraction rate (0.39 g·l^−1^·min^−1^) was at the highest enzyme loading of 0.05 ml·g^−1^.

#### 3.4.4 Effect of pH

The influence of pH on the extraction kinetics and the predicted equilibrium yields as summarized in and illustrated in Fig. 6(B_1_–B_3_), reveals that the highest predicted equilibrium yields for total carbohydrates and inorganic sulphates are at pH 5.25. For total dissolved solids it is at the more acidic conditions of the range, pH 4.5. Furthermore, the initial extraction rates for total carbohydrates and inorganic sulphates are highest at a pH of 5.25. The effect of pH on total dissolved solids' initial extraction rates are similar. From these results, it is clear that the mid-range of pH is more favorable for the extraction of polysaccharides in ultrasound assisted enzymatic extraction.

#### 3.4.5 Comparison between UAE, EAE and UAEE

**Fig. 8:**
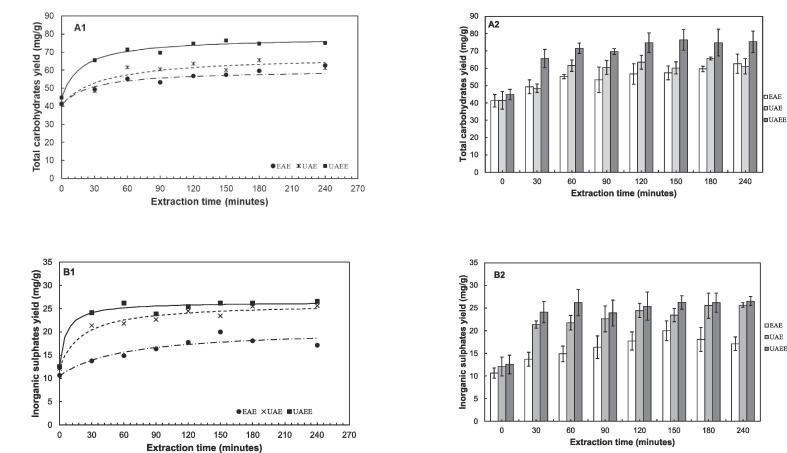
Comparison between EAE, UAE and UAEE on the extraction kinetics of total carbohydrates (A) and inorganic sulphates (B). Conditions (temperature, ultrasound intensity, enzyme dosage, pH) – EAE (52.5 °C, 0 W⋅cm^−2^, 0.025 ml⋅g^−1^, 5.25), UAE (52.5 °C, 59.16 W⋅cm^−2^, 0 ml⋅g^−1^, 5.25), UAEE (52.5 °C, 59.16 W⋅cm^−2^, 0.025 ml⋅g^−1^, 5.25). lines represents Peleg model kinetic curves.

The results of the evaluation of the relative impacts of EAE, UAE and UAEE on the yield of total carbohydrates and inorganic sulphates are summarized in Table 6 and illustrated in Fig. 8**,** and shows the observed yields at 240 min. ANOVA, followed by Tukey’s HSD post-hoc test (results not shown) confirmed that there were statistically significant differences in yields for total carbohydrates between the three methods (p-value < 0.05). However, for inorganic sulphates, there were no significant differences between UAE and UAEE, but there was a significant difference between EAE and UAE (or UAEE).

**Table 6:** Comparison of kinetic parameters and experimental yield data between enzyme-assisted extraction (EAE), ultrasound-assisted extraction (UAE) and ultrasound-assisted enzymatic extraction (UAEE).

**Method**	**Total carbohydrates**			**Inorganic sulphates**		
	**Initial extraction rate** (mg glucose·g^−1^·min^−1^)	**Predicted equilibrium yield** (mg glucose·g^−1^)	**Actual yield at 240 min** (mg glucose·g^−1^)	**Initial extraction rate** (mg SO_4_^2-^·g^−1^·min^−1^)	**Predicted equilibrium yield** (mg SO_4_^2-^·g^−1^)	**Actual yield at 240 min** (mg SO_4_^2-^·g^−1^)
EAE	0.71	57.81	62.62 ± 5.49^a^	0.17	20.67	17.12 ± 1.52^c^
UAE	0.72	67.91	61.11 ± 4.48^a^	0.67	26.13	25.63 ± 0.53^d^
UAEE	1.83	78.19	75.29 ± 6.24^b^	2.31	26.43	26.54 ± 1.03^d^

1 Different letters in the superscripts within a column indicates that the measured values are statistically different (*p < 0.05)*.

At 240 min, the yields for total carbohydrates were 62.62 ± 5.49, 61.11 ± 4.48 and 75.29 ± 6.24 mg glucose equivalents ·g^−1^ for EAE, UAE and UAEE respectively. For inorganic sulphates, the yields were 17.12 ± 1.52, 25.63 ± 0.53 and 26.54 ± 1.03 mg SO_4_^2-^·g^−1^ for EAE, UAE and UAE respectively. At 240 min extraction time, there were statistically significant differences in total carbohydrate yields between UAEE and both UAE or EAE, however, there was no statistically significant difference between UAE and EAE. For the yield of inorganic sulphates at 240 min, the yields for UAE and UAEE were similar, however, there was a significant difference between EAE and both UAE and UAEE.

## 4 Discussion

The work investigated the impact of extraction conditions on biomass solubilisation, and extraction of total carbohydrates and sulphated polysaccharides from South African kelp *Ecklonia maxima,* using a combined ultrasound-assisted enzymatic extraction. Extraction conditions were optimized, and an empirical kinetic model was fitted to better describe time-dependent behaviour of the combined system.

The findings portrayed that both EAE and UAE are effective methods in extracting total carbohydrates and thus fucoidan from the seaweed matrix, but UAE is more effective than EAE in extracting fucoidan as high yield of fucoidan are attained in a short period of time. The combined UAEE had improved extraction of total carbohydrates compared to both UAE and EAE, but did not result in improved extraction of fucoidan compared to UAE. However, both UAEE and UAE had higher extraction yields of fucoidan after 240 min compared to EAE. The differences in the impacts of UEA, EAE and UAEE on total carbohydrates and inorganic sulphates extraction could possibly be explained by the differences in the mechanism of each extraction method. The high-value polysaccharides in seaweed are part of the extracellular matrix, made up polysaccharides (alginates, fucoidans) as well as hemicellulose and celluloses [42]. The enzyme cocktail employed in this work selectively targets hydrolysis of the cellulose and hemicellulose causing perforation of the cell wall, and ultimately the release of polysaccharides and solubilization of other cell wall components [13], [43], [44], [45].

Ultrasonication facilitates in the physical breaking down and perforation of cell walls through intense localized cavitation which, leads to faster solubilization (as evident from Fig. 8) of polysaccharides from the cell wall matrix and within the cell wall. The differences in mechanisms likely explain the improved carbohydrate extraction when enzymatic hydrolysis was combined with ultrasound. These findings are in agreement with other studies where UAEE was used, and authors, generally agreed that the improved polysaccharides yields are due to complementarity of cavitation and enzymatic hydrolysis. Ultrasonication leads to physical particle breakdown, increased contact between enzymes and substrate, increased frequency of enzyme-substrate collision, and thus reduction in mass transfer limitations, leading to enhanced enzymatic hydrolysis [46]. The differences in impacts are validated by the kinetic analysis, which showed that initial extraction rates were higher when ultrasonication (for both UAE and UAEE) was applied compared to EAE.

From the results, it is evident that all the process parameters played a role to a certain degree in the extraction of total carbohydrates, inorganic sulphates and the solubilization of seaweed materials. Increasing temperature generally led to higher extraction of total dissolved solids, total carbohydrates, and inorganic sulphates, although, for the yield of total carbohydrates and inorganic sulphates, a maximum was reached, after which the yield declined. At higher temperatures, the viscosity, surface tension and density of water are reduced. The reduction in physio-chemical properties of water leads to deeper and easier penetration of water into the plant matter, which ultimately leads to better dissolution of solutes. Similarly, the increase in temperature leads to an increased enzymatic hydrolysis rate which could potentially explain the increase in the yields of total dissolved solids and total carbohydrates [34], [47]. Differently, it was noted that the increase in temperature eventually led to lower yields of solubilized materials. This is probably due to the denaturing effect of temperature on the enzymes [14], [47]. The enzyme cocktail, Accellerase 1500® is easily denatured at temperatures around 70 °C.

Ultrasound intensity also plays a pivotal role in the extraction process. Higher ultrasound intensities are linked with increased formation and collapse of cavitation bubbles which facilitate cell wall breakdown and solvent penetration that increases mass transfer rate. In this study, total dissolved solids, total carbohydrates, and inorganic sulphates yields all increased with increasing ultrasound intensity, approaching a maximum, after which stagnation or decline in yields was observed. For both total carbohydrates yield and inorganic sulphates yield, values increased until a certain level (up to 60 w·cm^−2^ and 80 w·cm^−2^ for total carbohydrates and inorganic sulphates respectively), beyond which an increase in intensity led to declining yields However, total dissolved solids did not exhibit a similar trend and values continued to increase with increased ultrasound intensity (Fig. 2 (A and D)). The decline in yields of total carbohydrates and inorganic sulphates are not fully explained, but could be due to degradation of these compounds at long exposure to high-intensity ultrasound, as has been shown previously for total carbohydrates[46].

Enzyme dosage played an important role in all response variables, although higher enzyme dosage did not necessarily result in higher recovery for all measured variables. From the response surface analysis, increased enzyme dosage resulted in increased yields of total dissolved solids and total carbohydrates. Even though this effect was not statistically significant during ANOVA analysis, this observation would be in line with the expectation that increasing enzyme dosage will increase the degree as well the rate of hydrolysis [47]. Total carbohydrates account for the solubilized sugars including the hydrolyzed oligosaccharides (both reducing and neutral sugars) from the cellulose and hemicellulose, whilst total dissolved solids account for total matter dissolved. The impact of enzyme dosage on fucoidan recovery is not clear, as it seems to increase to a maximum value at a specific enzyme dosage, and then decreases at higher enzyme dosages Fig. 4 (B and D). As fucoidans are interwoven with cellulose, hemicellulose and proteins within the cell wall matrix [13], [48], increased hydrolysis of the cell wall could be expected to increase fucoidan liberation, but this effect is not seen in this work. Enzymatic hydrolysis therefore does not seem to be very selective towards fucoidan extraction, even though it does result in carbohydrate hydrolysis and recovery.

The impact of pH on the process is complex, as it affects multiple phenomena that could lead to increased or decreased extraction of different compounds. Firstly pH affects enzyme activity[49], and Accellerase 1500® is denatured below the pH of 3.9 and above the pH of 7. Therefore, the results as illustrated in Fig. 3 (c), where lower amounts of carbohydrates are extracted, are expected as the pH extremes coincide with lower enzyme activity. Additionally, pH affects the solubility of different types of carbohydrates differently: the solubility of alginates typically increase with increasing pH, whereas conventional extraction of fucoidan is performed at low pH values[14]. For inorganic sulphates, there seems to be an increase in extraction as pH increases from the minimum pH towards some optimum, followed by a decrease. This may be explained by the solubility of alginate increasing with increasing pH, and possibly co-extraction of fucoidan up to a point where fucoidan solubility starts decreasing as pH increases further. This aspect requires further investigation, as definitive data were not generated in this investigation.

The results presented by RSM, and ANOVA are only useful in identifying optimum conditions for process parameters at the endpoint of extractions, but provides no information on the process evolution with time. Kinetic modelling provides the time dependent information, and can assist in determining appropriate processing times.

The Peleg model provided an adequate fit to the extraction data, giving high R^2^ values ranging from 0.70 to 0.98, 0.89–0.98 and 0.75–0.99 for total dissolved solids, total carbohydrates, and inorganic sulphates respectively. From the kinetic profiles (as illustrated in Fig. 7), total carbohydrates and inorganic sulphates portrayed similar trends, characterized by a high initial extraction rate, followed by a much slower phase associated with a gradual increase in yields until equilibrium is reached. This trend is in line with literature where various bioactives are extracted from plant matrices [37], [38], [50]. The high initial extraction rate is associated with the washing phase, which is the dissolution of the soluble fractions from the surfaces of the seaweed into the solvent, whereas the slow extraction phase is associated with diffusion-controlled transfer of solutes from the seaweed matrix into the solvent[37].

From the results in sections 3.2.1 − 3.4.4, it is clear that the mid-range of temperature (52 °C) seems favorable for UAEE, specifically for total carbohydrates and inorganic sulphates, as the highest values for these response variables were estimated at these conditions. This is in line with intuition, considering this region is potentially around the optimum temperature for the enzyme cocktail, thus the rate of hydrolysis at this temperature will be higher. Concerning the impact of ultrasound intensity, it was evident that the application of ultrasound increases the initial extraction rates and extent of extraction as opposed to no ultrasound. This is expected since the cavitational effect of ultrasound probably dominates the extraction as opposed to the slower enzymatic hydrolysis mechanisms where no ultrasound is applied. Interestingly, the highest ultrasound intensities did not have the highest initial extraction rates, rather the mid-range (59 W·cm^2^) had the highest rates for total carbohydrates (1.83 mg·g^−1^·min^−1^) and inorganic sulphates (1.50 mg·g^−1^·min^−1^). It is expected that increasing intensity will increase extraction rates as cavitational effects increase, however, literature has also pointed out that increasing intensity may often result in poorer ultrasound propagation in medium, thus less intense cavitation. This could probably explain the decline in extraction rates at the highest ultrasound intensities employed. Noteworthy, the highest extent of extraction for total carbohydrates was at the highest intensity, the kinetic profile (Fig. 7 (B)) shows a steady increase in total carbohydrates (with the rate not declining as in other experimental conditions). This could potentially show that at higher intensity, cavitational effects may be lower, but there is higher enzymatic activity, which could potentially explain the increase in total carbohydrates. Literature has pointed out that ultrasound and enzymatic hydrolysis could be complementary, however, we need to be mindful of how ultrasound may affect the enzyme’s activity [33].

## 5 Conclusions

This study explored the potential of ultrasound-assisted enzymatic extraction (UAEE) for extracting fucoidan from *Ecklonia maxima*. Notably, UAEE outperformed both enzyme-assisted extraction (EAE) and ultrasound-assisted extraction (UAE) in terms of total carbohydrate yield, displaying a 20 % increase after 240 min.

The kinetics analysis revealed that UAEE and UAE had similar profiles and higher yields of inorganic sulphates compared to EAE, with a remarkable 60 % improvement after 240 min. However, the use of ultrasound in conjunction with enzymatic hydrolysis did not exhibit any advantages in terms of fucoidan extraction yield.

Optimization through desirability functions pinpointed optimal conditions for maximum yields: a temperature of 52.5 °C, ultrasound intensity of 59.17 W·cm^−2^, enzyme dosage of 0.0375 ml·g^−1^, and pH 5.25, resulting in predicted yields of 79.19 mg glucose equivalents⋅g^−1^ and 25.46 mg SO4^2-^⋅g^−1^ for total carbohydrates and inorganic sulphates, respectively. When focusing solely on inorganic sulphates, an optimal condition of 58.75 °C, ultrasound intensity of 88.75 W·cm^2^, enzyme dosage of 0 ml·g^−1^, and pH 6 yielded 23.74 ± 1.52 mg SO_4_^2-^⋅g^−1^, equivalent to 7.9 % fucoidan (w/w) from the dry seaweed.

In conclusion, this research demonstrates the potential of UAE, EAE, and UAEE as environmentally friendly approaches for seaweed polysaccharide extractions. Among these methods, ultrasonication holds promise for fucoidan extraction. The results from this study, could provide valuable data for process design and scaling-up of extraction system, in the context of kelp volarization.

## CRediT authorship contribution statement

**Zwonaka Mapholi:** Conceptualization, Data curation, Formal analysis, Investigation, Methodology, Validation, Writing – original draft, Writing – review & editing. **Neill Jurgens Goosen:** Conceptualization, Writing – review & editing, Funding acquisition, Resources, Supervision.

## Declaration of competing interest

The authors declare that they have no known competing financial interests or personal relationships that could have appeared to influence the work reported in this paper.
